# Fulminant Necrotizing Pyomyositis Tropicans

**DOI:** 10.7759/cureus.21767

**Published:** 2022-01-31

**Authors:** Snehasis Das, Oseen Shaikh, Naveen Kumar Gaur, Chellappa Vijayakumar, Uday Kumbhar

**Affiliations:** 1 Surgery, Jawaharlal Institute of Postgraduate Medical Education and Research, Puducherry, IND

**Keywords:** immunocompetent, septic shock, debridement, antibiotics, pyomyositis

## Abstract

Pyomyositis tropicans is a purulent invasive infection of the striated muscle tissues, usually caused by Gram-positive bacteria *Staphylococcus aureus* and Streptococcus in immunocompromised patients. We hereby report a case of fulminant necrotizing pyomyositis that occurred in a 16-year-old immunocompetent patient, and it is the first one of its kind to the best of our knowledge. The patient underwent imaging which suggested extensive intramuscular abdominal wall abscess formation, for which the patient underwent multiple surgical debridements of the lateral thoracic wall. Subsequent cultures grew *Escherichia coli* as the causative organism. Postoperatively, the patient went into catastrophic, irreversible septic shock ending in an eventual fatality.

## Introduction

Pyomyositis tropicans is an invasive infection of the striated muscles with Gram-positive organisms heralding its causation. A rare lethally fatal necrotizing variant reported from *Escherichia coli* (*E. coli*) has been sparsely reported, which is postulated to affect mainly the immunocompromised patients [1,2]. With less than 20 cases reported in the literature, the hematogenous spread has been documented as the predetermining factor for the unusual systemic dissemination of the disease with patients presenting with florid septic shock. Early debridement and culture-based antibiotic therapy have been seen to offer a fighting chance in an otherwise lethal ailment. With this case, we report the first instance of this rare entity affecting an immunocompetent individual resulting in deadly sepsis and eventual demise.

## Case presentation

A 16-year-old female with no comorbidities presented with complaints of pain in the left flank and obstipation for two days. The patient had a history of multiple episodes of non-bilious vomiting with intermittent low-grade fever, which subsided with medications. She gave a history of swelling in the left perianal region, which started around 12 days back and ruptured spontaneously over the next two days to discharge pus, for which they had shown to a local hospital. The wound was subsequently dressed at a local hospital. The patient turned lethargic over a week and was brought to the hospital after the above complaints. There was no prior history of similar complaints in the recent past or childhood.

The patient had a blood pressure of 130/90 mmHg, pulse rate of 156 beats/min, and respiratory rate of 35/min but was maintaining a saturation of 96% at room air. Generalized abdominal distension was noted with an absence of bowel sounds. No specific areas of tenderness or guarding were made out. An ill-defined, deep-seated, tender swelling of around 10 cm × 10 cm was made out in the left lumbar and paraspinal region extending from the level of the 11th rib to the level of the iliac crest. The skin over the part of swelling was warm, but no other changes could be made out. The site of the previously ruptured left perianal abscess contained granulation tissue with no active pus discharge.

**Figure 1:**
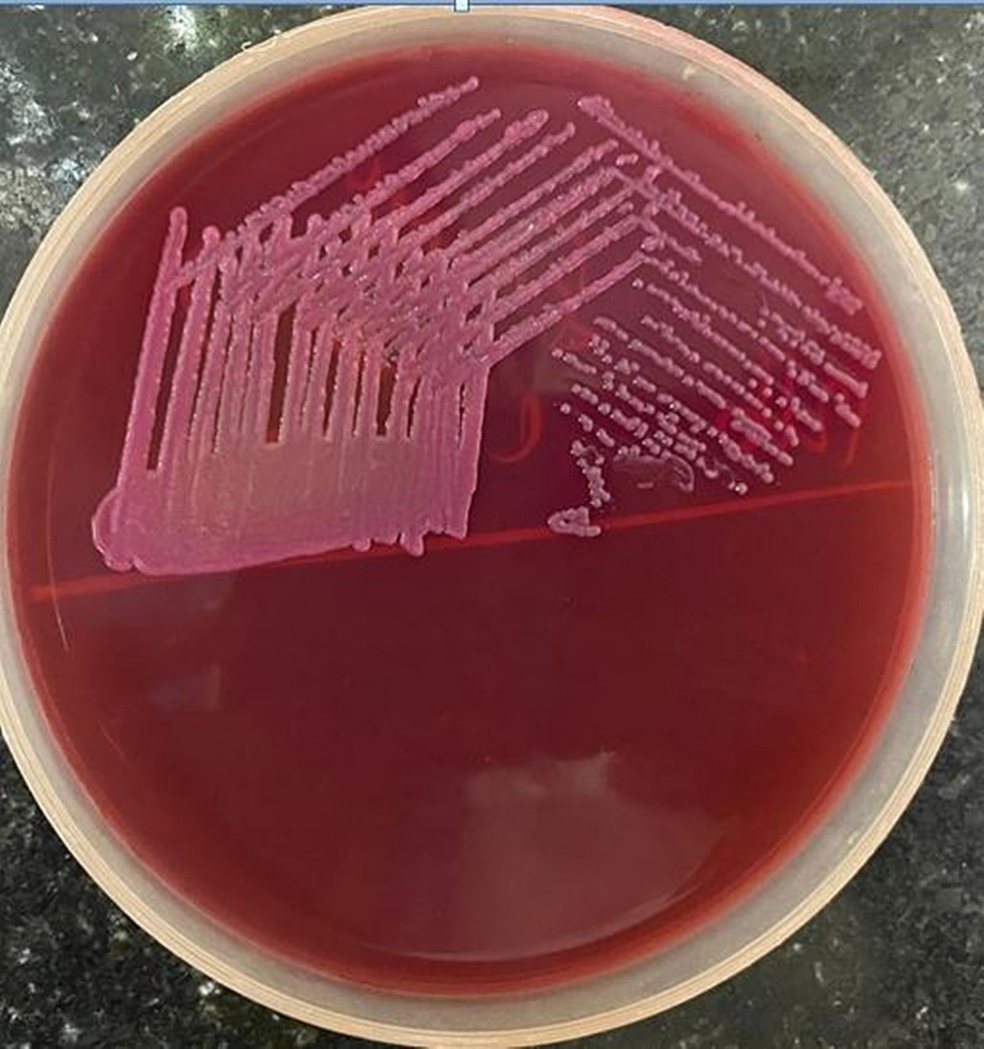
Plating of the organism from the blood culture showing colonies of the Escherichia coli.

Blood investigations showed the patient having moderate anemia and leukocytosis of 14,800 cells/mm^3^. The liver function test (LFT) and renal function test (RFT) were normal. Sequential blood cultures grew *E. coli *sensitive to ceftazidime and *E. coli* with *Enterococcus faecium* sensitive to meropenem (Figure 1).

**Figure 2:**
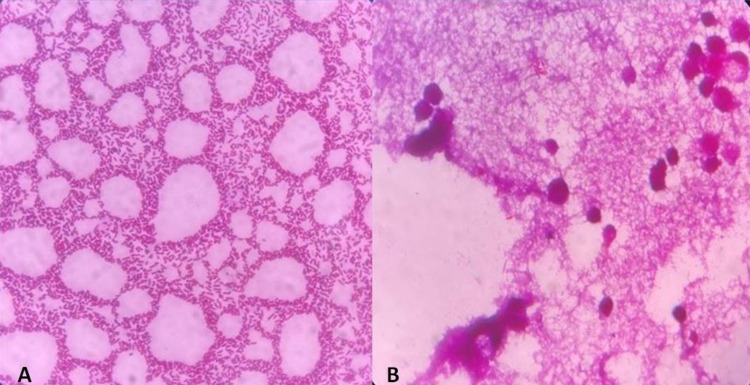
A) Gram staining of the exudates from the pus having Gram-negative rods and B) the necrotic muscle having Gram-negative rods and pus cells.

Intraoperative muscle biopsy and culture showed fibroadipose tissue and skeletal muscle fibers with acute inflammatory infiltrate and necrosis suggestive of pyomyositis. The culture showed the growth of Gram-negative rods with pus cells (Figure 2).

**Figure 3:**
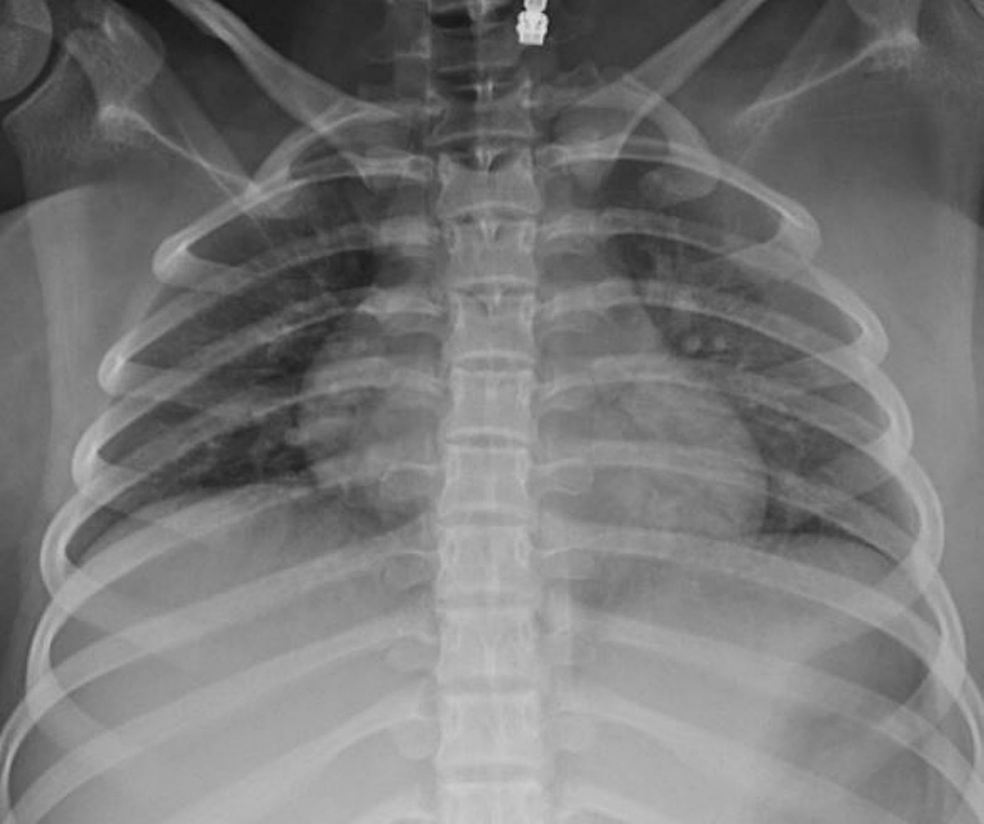
Chest x-ray showing no evidence of any infectious origin or pleural effusion.

Chest x-ray of the patient revealed normal bilateral lung fields (Figure 3). Abdominal x-rays revealed dilated small and large bowel loops.

**Figure 4:**
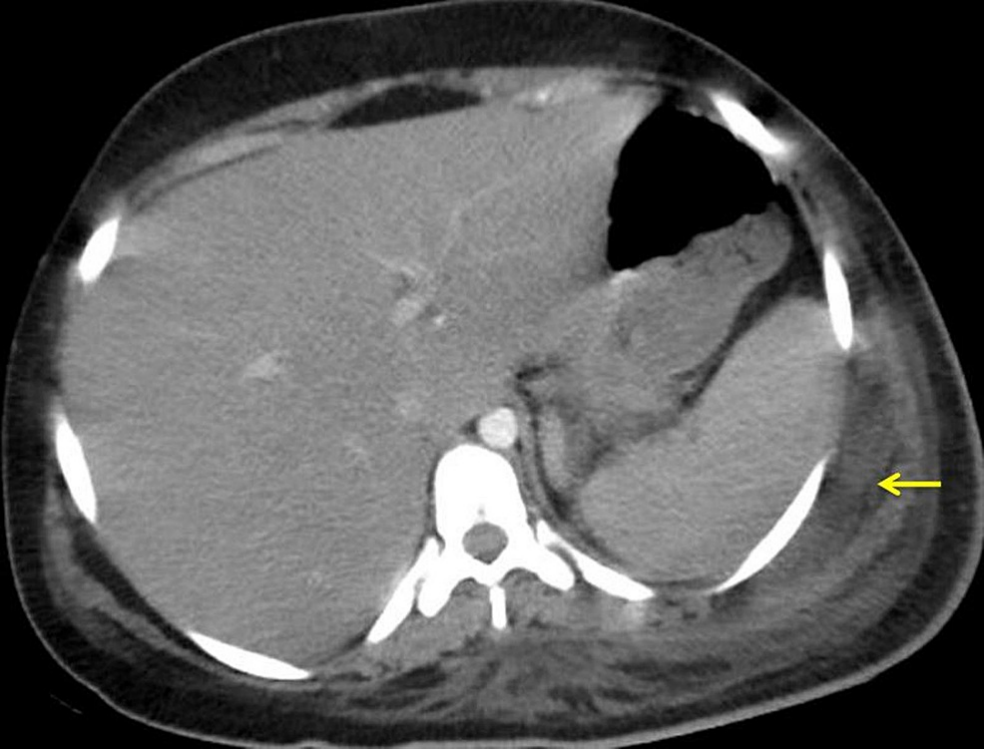
CT (axial view) abdomen showing evidence of intramuscular and submuscular pus (arrow) with fat stranding around the muscle in the left flank region.

Abdominal ultrasound showed few small bowel loops with sluggish peristalsis of maximum caliber 3.6 cm in right iliac fossa with no evidence of any mechanical obstruction. Contrast-enhanced CT abdomen and thorax showed grossly dilated small bowel loops, fluid-filled with a maximum caliber of 4.6 cm in the hypogastric region, and showed normal bowel wall enhancement. No hypodense/irregular bowel wall thickening was noted. The caecum and ascending colon were also dilated with a maximum caliber of 4.2 cm. The rest of the large bowel loops were collapsed. The ill-defined hypodense collection was seen along posterior, lateral thoracic, and abdominal walls on the left side, with a maximum width of 18 mm (Figure 4).

At presentation, the patient had metabolic acidosis and acute kidney injury. The patient was resuscitated with intravenous fluids, analgesics, and antibiotics. Bedside aspiration of the previously defined swelling was done, which revealed purulent fluid at the level of lateral abdominal wall muscles. CT with rectal contrast was obtained which showed dilated small bowel and large bowel loops until the transverse colon level with collapsed descending colon segment. No active extravasation of contrast was noted at the transition.

**Figure 5:**
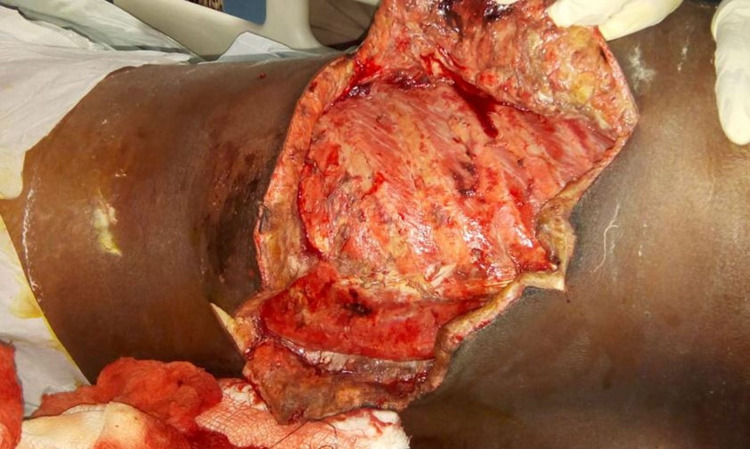
Postoperative wound over the left flank and back region after debridement.

The patient was started empirically on higher antibiotics and then changed to ceftazidime and meropenem based on blood culture and pus culture. The patient did not improve clinically. Hence patient was planned for debridement under general anesthesia to drain pus collections in the subcutaneous and intramuscular planes of the left posterior thoracoabdominal wall. Intraoperatively, we found that multiple muscles like infraspinatus, rhomboids, latissimus dorsi, and the deeper layers of levator spinae and erector spinae were necrosed. All the necrosed tissue was debrided (Figure 5).

Postoperatively, the patient was not extubated. The patient became hypotensive and developed severe acute kidney injury, requiring hemodialysis. However, the patient did not show any signs of improvement and succumbed to death due to multiorgan dysfunction syndrome.

## Discussion

Pyomyositis tropicans is an acute bacterial infection of the skeletal muscle system with its clinical presentation closely related to necrotizing fasciitis. It is usually caused by Gram-positive organisms, namely *Staphylococcus aureus*, and S*treptococcus pyogenes*, in a limited spectrum of people who are immunocompromised, affecting mostly malignancy, transplant, and intensive care unit (ICU) patients. *E. coli* is the most common organism responsible for Gram-negative bacteremia, with documented cases of fatal sepsis [1]. Fulminant necrotizing myositis caused by *E. coli* is a rare clinical entity that has slowly come to existence affecting only immunocompromised patients with grave mortality of around 80% to 100%.

The first case of *E. coli-*induced pyomyositis was documented in 2009, affecting a patient with acute myeloid leukemia [2]. Initial research on this ailment revealed a predilection for immunocompromised patients with a male to female ratio of 3:1 [3]. The common risk factors included patients suffering from malignancy, diabetes mellitus, obesity, cirrhosis, chronic renal insufficiency, intravenous drug abusers, and in rare instances, trauma was also reported [4]. In tropical countries, it has been seen to occur synergistically with tissue parasites favoring the extremities, whereas, in temperate countries, it tends to occur more in the extremities.

The clinical spectrum of this syndrome ranges from common muscle pain that is universally reported to frank refractory septic shock affecting multiple organ systems in the body, as happened in our case. Pathophysiology takes the disease through three notable stages [5]. Stage I, named the invasive stage, would involve only starting muscular inflammation with no pus yield on aspiration. Stage II, named the purulent stage occurring at the second week and the third week, would present with definitely localized abscess formation with no evidence of systemic dissemination. Stage III is also called the toxic stage and would present multiple organ systems failure with septicemia and shock. Most of the patients present to the hospital at stage II, from where a judiciously and clinically treated patient will have a fair chance of survival. In contrast, stage III was known to have a universal fatality, as was seen in our case [5]. Our patient had presented in stage III and had organ dysfunction.

Ultrasonography of the locally affected site followed by a quick CT scan of the body indicates the first radiological clues to the disease. Plain radiographs would be normal and would hence be of no use in this matter. Ultrasonography, if positive, is used for image-guided aspiration of the abscesses and is immediately sent for culture sensitivity [6]. CT scans can help envisage findings of heterogeneous attenuation of enlarged, inflamed muscles with a focal rim of enhancement around them [7]. In case of failure of all these radiological investigations, MRI can be used to reveal inflamed fascial planes with infected compartments and surrounding soft-tissue fat-stranding [8]. Our patient had undergone CT, which showed an ill-defined collection over the left lateral thoracic and abdominal wall. We did not do MRI on our patient, as the patient’s condition deteriorated rapidly.

Aggressive management with broad-spectrum antibiotics at the start followed by culture-specific targeted high-dose therapy is the dictum. The patient is usually taken up for immediate guided drainage of the pus collections, failing which, incision and drainage are done under general anesthesia. This organism is studied to have intrinsic resistance from fluoroquinolones. As analyzed by multiple cohorts was found to be *E. coli* sequence type 131 (ST131) [9,10]. The clinical progression is quite aggressive, with the transition between stages occurring in days and an exponential increase in mortality with each up-gradation. The antibiotics course and the source debridement should continue until the patient shows some degree of clinical and radiological improvement. This improvement is seen to take weeks to months with a thunderous course of action, mainly in the ICU setup with round-the-clock monitoring. Otherwise, it might result in imminent mortality in the case of advanced disease with pan systemic involvement with frank sepsis shock.

In our case, we report the first of a kind, an atypical rare clinical syndrome of fulminant necrotizing pyomyositis caused by *E. coli* affecting a young immunocompetent individual in stage III at presentation. Despite aggressive antibiotic usage and debridement, the patient did not improve and expired due to multiorgan dysfunction syndrome.

## Conclusions

*E. coli*-associated fulminant necrotizing pyomyositis is a rare ailment in the medical field and could veil a potentially life-threatening clinical outcome for which it requires a high degree of suspicion for diagnosis. Bearing a fatal prognosis with a rapid transition between clinical stages requires ICU monitoring with multiple aggressive debridements and targeted antibiotics coverage. Even with near-total mortality rates in stage III with septic shock, early intervention with close monitoring of blood parameters and culture-specific antibiotics might give the patient a fighting chance. Previously not described in the literature, we establish that this syndrome affects the immunocompetent with the same fatality and requires a high risk of suspicion for prompt diagnosis and treatment.
